# Transactions of the Medical Association of Southern Central New York

**Published:** 1849-03

**Authors:** 


ART. V.—*Transactions of the Medical Association of Southern Central New York. Convened at Ithaca, June,* 1848.

The formation of an Association for Southern Central New York, embracing the counties of Chemung, Tompkins, Tioga, Cortland, Broome and Cayuga, was noticed in a former number of this Journal. The Association has been instituted by some of the most eminent members of the profession in these counties, to promote interchange of professional views, and mutual improvement. The transactions of the meeting held in June last are just published, making a pamphlet of 16 pages. The meeting appears to have been well attended, and the proceedings promise well for the Association. Committees were appointed to prepare essays on voluntary subjects, and to report on endemics and epidemics in the several counties which the Association embraces. The plan seems to us admirable, and we should rejoice to see the example followed in other portions of the Empire State. If meetings of the County Societies generally are as barren in interesting or useful transactions as in some counties that we might mention, there is abundant room for improvement, and it may be worthy of consideration whether the union of the profession of several contiguous counties, to form a single association, will not be preferable to the present system of a distinct society for each county. The publication of the transactions of medical societies is desirable, as a means of diffusing valuable information, and as an inducement for individuals to contribute the results of their observations and reflections. If the plan, in this respect, of the Association of Southern Central New Yoik were to be adopted generally throughout the State, the annual information concerning endemic and epidemic diseases in different sections, would, alone, be highly valuable. We beg leave to commend this subject to the consideration of our medical brethren, not only in this State, but in sister states.

We quote from the 'Transactions' the following digest of an essay on *Lumbar or Psoas Abscess*, submitted by Dr. Hyde, of Cortland:—

"Dr. Hyde, of Cortland, next read a very full essay on lumbar, or psoas abscess. He referred to the results of his observation of some 30 cases which had occurred in his own practice, and those of his professional neighbors within a circuit of about thirty miles, and during a period of 13 years. The disease had occurred in patients between the ages of 18 and 70 years, in a majority of instances, however, in those between 40 and 50 years of age. His conclusion from statistical examinations was, that cases of this affection were far more frequent in this and the New England States, than in the southern portions of our country. Of the whole number of cases which he had observed, five only were females, and but one of these exhibited the physical conformation recognized as the scrofulous diathesis. Of the 25 males who had the disease, two only gave evidence of possessing the strumous habit, while the remaining number were of vigorous constitution; affording no suspicion either of scrofulous or tubercular predisposition. The period of the disease varied from two months to seven years. The majority of the cases were of about three years' duration.

The earliest *symptoms* of the affection were rarely observed, since the patients seldom presented themselves for professional advice, until such severe structural changes had taken place, as to render a diagnosis of little value as a basis for successful treatment. There was, however, a marked want of identity in the earlier symptoms that were observed, so much as to embarrass the arrival at correct conclusions, even so late as the external pointing of the abscess. Post mortem examinations, however, showed an identity of pathological conditions, both of the original structures involved, and the results of the diseased process. As to the etiology of the disease, the writer was fully of the opinion, that, in the instances cited, at least, it originated in a concurrence of accidental causes, rather than from any predisposition to it, identical with the physical organization; believing, however, that similar causes would more readily excite the disease in one of a strumous habit, than in one possessing an originally sound condition. *Pathology.*—Of the thirty cases observed, nine were examined post mortem. In only one instance did he find disease of the spinal column. The result of these examinations forced upon him the conviction that the structures originally affected by the morbid process, were, either the psoas muscle, or its immediate envelops; and that cases which show an alteration of vertebral structure, are instances of tissues involved by the extension of the original disease. The *prognosis* is decidedly unfavorable. The writer had never witnessed a single recovery in a well marked case of the disease. Several cases gave strong promise of ultimate recovery, but eventually the disease returned with exasperated symptoms and terminated fatally. It was suggested that the *diagnosis* of the disease in its earlier stages might be made by observation of the deranged function of the psoas muscle, in reference both to the movement of the body and extremity, the position of the limb in relation to the body, both in erect and recumbent postures, and by attentive consideration of the pain when compared with that of real lumbago. The *treatment* that gives any promise of success, must be applied in the earliest inflammatory stage. When pointing took place, the writer recommended the evacuation of the abscess after the method of Prof. MILLER, of Edinburg."

The conclusion of Dr. Hyde, as respects recovery, we suspect to be more unfavorable than a larger number of observations would warrant. The disease, it is true, will prove fatal, sooner or later, in a large proportion of cases, yet recovery not unfrequently takes place, especially if the spinal column be not involved, and the subject be not of a scrofulous diathesis. Two instances of a favorable result were communicated at the monthly meeting of the Medical Association of this city in January last, and a third at the meeting in February; in the latter case the affection having been of long duration, and the spine evidently implicated.

We do not profess to have had much practical acquaintance with the disease, but circumstances have led us to bestow upon it, of late, anxious reflection; and we are disposed to think that confinement and quietude, together with antiphlogistic treatment, are often far from promoting a cure. The chief source of danger is the exhaustion produced by protracted purulent evacuations; and, it is to be considered, that, if not occurring in connection with scrofulosis, it is apt to be associated with an cachectic habit. The indications of treatment, under these circumstances, are, first, to improve the constitutional vigor by tonics, nutritious diet, fresh air, and moderate excercise; and, second, to restrain, as far as possible, the formation of pus. The general remedies mentioned under the first head, are appropriate to the second, but, in addition to these, the local measures recommended by Prof. Brainard, of Chicago, viz: injections into the cavity of the abscess of astringent fluids, deserve trial. In a large proportion of cases, when the character of the disease is first ascertained, whatever inflammatory action may have existed, has ceased to exist. The treatment which might have been due to an inflammation, is, therefore, now not called for. We have only the products and consequences of the inflammatory action, and to carry the disease to a successful termination, it is necessary to preserve and strengthen the power of the system, so that it may triumph by its endurance and sanative energies. We throw out these few ideas, and shall be glad if they provoke communications from some of our readers.

